# An overview of dementias

**DOI:** 10.4103/0972-2327.74245

**Published:** 2010-12

**Authors:** P. S. Mathuranath

**Affiliations:** Cognition & Behavioural Neurology Section (CBNS), Department of Neurology, Sree Chitra Tirunal Institute for Medical Sciences & Technology (SCTIMST), Trivandrum - 695 011, Thiruvananthapuram, Kerala, India. E-mail: mathu@sctimst.ac.in

This issue of the supplement of the journal focuses on dementia. A series of articles, many by the leading investigators in the field, covering a wide range of topics related to dementia, both clinical and theoretical issues are presented. This supplement focuses more on Frontotemporal lobar degeneration (FTLD), a clinical subtype of degenerative dementia that has traditionally remained in the shadows of Alzheimer’s disease. The collection of articles cover the entire spectrum related to dementia, starting from clinically diagnositic issues, such as an approach to non-degenerative causes of dementia to providing an indepth insight into the clinical issues related to Frontotempolar dementia and its subtypes of primary progressive aphasias, to a clear insight into the neurobiology and the pathogenetic mechanisms involved in Alzheimer’s disease, to providing an overview of one of the emerging vistas of gait and cognition and biomarkers in Alzheimer’s disease and finally providing a peek into some of the management aspects of dementia. A vast majority of articles give an overview of the progress made over the past one decade in that particular area.

It is my belief that this supplement will prove to be a handy source of reference for established practitioners in the field as well as for beginners, and also for students of Cognition and Behavioural Neurology. It will also make a useful reading for general neurologists and psychiatrists who would be interested in getting a bird’s eye view into some of the more intricate aspects of dementia.

